# Effects of Thickness Fraction Process on Physicochemical Properties, Cooking Qualities, and Sensory Characteristics of Long-Grain Rice Samples †

**DOI:** 10.3390/foods11020222

**Published:** 2022-01-14

**Authors:** Sara E. Jarma Arroyo, Terry J. Siebenmorgen, Han-Seok Seo

**Affiliations:** Department of Food Science, University of Arkansas, 2650 North Young Avenue, Fayetteville, AR 72704, USA; sejarmaa@uark.edu (S.E.J.A.); tsiebenm@uark.edu (T.J.S.)

**Keywords:** rice, thickness, fraction, grading, physicochemical, cooking, sensory

## Abstract

A process of removing thinner kernels of rough rice, i.e., thickness fraction process, has been suggested as a method for increasing milling yields in the rice industry. This study aimed at determining whether physicochemical properties, cooking qualities, and sensory characteristics of rice samples could be changed by the addition of a thickness fraction into the rice process stream. Each of four long-grain rice cultivar lots was assigned into two groups: unfractionated and thickness-fractionated. For the thickness-fractionated group, thin rice kernels (<1.9 mm) of rough rice were discarded from unfractionated rice samples. Unfractionated and thickness-fractionated rice samples were compared with respect to physicochemical properties, cooking qualities, and sensory characteristics. The results showed that the removal of such thin kernels decreased the breakage and chalkiness rates and increased head rice yields. Fractionated rice samples exhibited lower amylose contents and crude protein contents but higher gelatinization temperatures than unfractionated rice samples. While the optimum cooking duration and width–expansion ratios of thickness-fractionated rice samples were higher than those of unfractionated ones, there was a negligible impact of the thickness fraction process on sensory characteristics of long-grain rice samples. In conclusion, the thickness fraction process affects physicochemical properties and cooking qualities more than the sensory characteristics of rice samples.

## 1. Introduction

Thickness fraction, also known as thickness grading, of rough rice, refers to a process through which rough rice is first screened according to size into different thickness fractions, and the thinner kernels are then removed for other applications such as flour or parboiling [1]. Thickness fraction has been previously proposed as a process for improving the milling operation and reducing kernel breakage during the milling of rough rice [2]. Jindal and Siebenmorgen [3] showed that thicker kernels of rice produced dramatically greater head rice yield (HRY) compared to thinner kernels due to a higher susceptibility to moisture absorption of thinner kernels that may increase the kernel breakage rate during milling. Additionally, Chen et al. [4] observed that the thinnest rice kernels (<1.49 mm) presented a higher surface lipid content compared to the thicker kernels (>1.49 mm) after milling under identical conditions. These results suggest that the pressure to which rice is subjected during milling, or the duration of the milling procedure will result in the thinnest kernel fraction being milled at a greater bran removal rate than the other kernel fractions. Thinner kernels require a shorter milling duration. Thus, if thinner rice kernels are separated from thicker kernels and milled separately, breakage rates could be reduced, resulting in higher milling yields.

Thickness fraction was also shown to impact rice’s physicochemical properties [1,4], with thinner kernel fractions having greater amounts of crude protein and lipids and lower starch content than thicker kernel fractions [1,4]. Since starch is the predominant constituent of rice, the estimation of starch’s two main fractions, amylose and amylopectin, embodies a major cooking and sensory quality indicator. Rice with high amylose content (25–30%) tends to cook more firmly and with less stickiness, while rice with lower amylose content (<20%) tends to cook more softly and with greater stickiness [5]. The amounts of specific components such as protein and lipid also influence the cooking properties of rice [6,7]. More specifically, the protein content of rice was found to affect textural characteristics of cooked rice through competing with starch for water with the formation of disulfide bonds, while lipid restrains the moisture uptake of rice during cooking through the production of amylose–lipid complexes that impede leaching of amylose and swelling of starch [6]. It was demonstrated that such differences in physicochemical properties directly influence the cooking and sensory qualities of rice [7,8,9].

Since rice is usually consumed as an intact grain, rice industries tend to determine the economic value of rice in terms of cooking and eating qualities that can be measured in terms of water uptake ratio, kernel elongation during cooking, solids in the cooking water, cooking duration, and sensory characteristics [10]. The steady incremental growth of rice consumption is also accompanied by stricter consumers who demand rice products that exhibit premium qualities. Consumers consider grain appearance with respect to shape, size, or color to be the major attribute that determines the quality of rice [10]. For example, rice products, including many broken and/or chalky kernels (i.e., opaque grain regions), are considered to represent a low-grade quality that may in turn decrease overall market value [11,12], influencing consumers’ purchase decisions. After milling, rice colors vary from white to yellow depending on variety, pre-processing, and storage conditions, and consumers are overall more likely to favor white rice over yellowish rice [5,9]. Furthermore, the appearance, aroma, flavor, and texture are all sensory characteristics of cooked rice that can play a crucial role in consumer acceptance [13,14,15,16]. Meullenet et al. [17] indicated that the most important sensory characteristics influencing Asian consumers’ acceptance were appearance (esp., degree of whiteness and visual stickiness) and aroma/flavor (starchy, cooked grain, nutty, sulfur, heated oil, and metallic), and high cohesiveness, greater softness, and low stickiness were also found to be textural characteristics preferred by Asian consumers. However, it should be noted that non-sensory characteristics such as price, rice-growing location, and nutritional value are also considered to be factors that can influence consumers’ preferences and purchase decisions [17,18].

Even though the benefits of including a thickness fraction process in the milling quality of rice have been widely explored, little attention has been paid to the influences of thickness fractioning on rice’s cooking and sensory qualities. Since the inclusion of thickness fraction into the rice processing stream would inevitably lead to incremental cost increases for rice processors, the impact of the thickness fraction process in rice industries should be carefully evaluated. The primary objective of this study was, therefore, to determine whether cooking qualities and sensory characteristics, along with physicochemical properties, could vary if a thickness fraction step was added into the rice processing stream, i.e., comparisons between unfractionated and thickness-fractionated rice samples.

## 2. Materials and Methods

### 2.1. Rice Samples

Two long-grain pureline cultivars (Cheniere and V3501) and two hybrid cultivars (XL753 and XP760) grown in Harrisburg, AR, were included in this study. Each cultivar was harvested at a medium moisture content level (18.5–20.5%, wet basis) during fall 2015. Rough rice samples were cleaned using a dockage tester (XT4, Carter-Day, Minneapolis, MN, USA), and the cleaned lots were then conditioned to a 12.0 ± 0.5% (wet basis) moisture content using a climate-controlled chamber (26 °C and 56% relative humidity) regulated by a stand-alone conditioner (Model 5580A, Parameter Generation and Control, Black Mountain, NC, USA).

### 2.2. Thickness Fraction of Rough Rice and Milling Properties

As described above, each of four rice cultivar lots was assigned into two groups: unfractionated and fractionated. More specifically, in addition to bulk unfractionated rice, a portion of each bulk rice lot was thickness graded at a 12.0 ± 0.5% (wet basis) moisture content using a precision sizer (Model ABF2, Carter-Day, Minneapolis, MN, USA) equipped with rotary screens (30 cm diameter). Bulk rice was screened only once and split into two thickness fractions, i.e., thin (<1.9 mm) and thick (≥1.9 mm), and for the purposes of this study, the thin fraction was discarded. In a preliminary test aimed at determining the cutoff levels of thickness grading, a total of 123 rice consumers rated overall difference levels between unfractionated rough rice and each of five thickness-fractionated rough rice samples: 1.6 mm, 1.7 mm, 1.8 mm, 1.9 mm, and 2.0 mm on 10-point scales ranging from 0 (no difference) to 10 (extreme difference) [16,19]. Each sample was presented in a glass bowl (118 mL) identified with a three-digit code. Because there were no significant differences among the rough rice samples varying in thickness, a standard thickness cutoff of 1.9 mm was chosen based on previous research [20,21,22] to mimic what could occur in a realistic setting in industry.

The thicker kernels (≥1.9 mm), referred to here as fractionated or thickness-graded rice and the unfractionated rice portions were used for further analysis. Each fraction was weighed to determine the mass distributions of the bulk rice. Rough rice representing each cultivar was dehulled using a dehusker (THU-35, Satake, Hiroshima, Japan), and the resultant brown rice was milled using a laboratory miller (McGill number 2, RAPSCO, Brookshire, TX, USA). In order to develop relationships between the degree of milling (DOM) and milling duration, the rice was milled for durations of 10, 20, 30, and 40 s to determine the milling duration required to achieve the desired DOM, a 0.40 ± 0.05% surface lipid content (SLC), for each lot and fraction. Each rice sample was stored in a sealed container at 4 °C that was stored at room temperature (22 °C) for 24 h prior to sample preparation and further analysis. Head rice yield was determined, with unbroken heads separated from broken kernels using a sizing device (Model 61, Grain Machinery Manuf. Corp., Miami, FL, USA) and retained for determination of quality properties.

### 2.3. Physical Properties

In order to determine the effects of thickness fraction on the physical properties of rice, approximately 100 kernels of head rice were weighed and placed in a 32 mm-thick tray (152 mm × 100 mm × 20 mm), with no two kernels allowed to be in contact. Kernel dimensions, broken numbers of kernels, area of chalky kernels, and the discoloration of head rice samples were measured using an image analysis system (SeedCount SC 5000, Next Instruments, Condell Park, Australia). From a preload profile, kernel discoloration was defined in terms of a discolored area percentage of yellow, red, brown, black, green, and light pink shades. Chalky areas of milled rice (100 kernels of each cultivar/fraction/replicate) were determined using the procedure of Ambardekar et al. [23]. After kernels were scanned, the software determined the number of color pixels associated with both non-chalky and chalky tissue, previously identified through a calibration procedure, and calculated the percentage of chalky total kernel area. The degree of whiteness (L*) was also determined by scanning approximately 60 g of head-rice kernels by using a near-infrared reflectance (NIR) spectroscopy (NIR-DA 7200, Perten Instruments, Huddinge, Sweden). All analyses were evaluated in triplicate.

### 2.4. Chemical Properties

In order to determine the effects of thickness fraction on amylose content, crude protein content, moisture content, pasting properties, and thermal properties of rice samples, a 60 g portion from one head rice sub-sample was ground into flour using a cyclone mill (3010-30, UDY, Fort Collins, CO, USA) with a 0.5 mm screen.

Amylose content was determined using the simplified iodine assay method of Juliano [24]. Approximately 100 mg of rice flour were transferred into a 50 mL test tube to which 1 mL of 95% ethanol and 9 mL of 1 N NaOH was added, after which the sample was heated for 20 min in a boiling water bath. After cooling, the content was transferred into a 100 mL volumetric flask, and its volume increased up to 100 mL. A 5 mL aliquot was then pipetted from the 100 mL solution into a disposable test tube, and 0.1 mL of 1 N acetic acid and 0.25 mL of iodine solution were added. The mixture was then stirred and allowed to incubate for 30 min, after which the absorbance was read at 620 nm. The amylose content of the sample expressed on a percentage basis was determined by reference to a standard curve. The crude protein content of milled-rice samples was ultimately determined by scanning approximately 60 g of head-rice kernels using NIR spectroscopy (NIR-DA 7200, Perten Instruments, Huddinge, Sweden). The moisture content of the rice flour was measured according to the AACC International method (44-15.02) [25], i.e., by drying a 2.5 g portion in a 130 °C oven for 2 h. All analyses were evaluated in triplicate.

The pasting properties of the rice samples were determined using a Rapid Visco Analyzer (RVA Super 4, Newport Scientific, Warriewood, Australia). Approximately 3 g of flour were combined with 25 mL of deionized water in an aluminum cylinder, with the exact quantities of rice flour adjusted according to sampling MC at a 12% moisture basis. The flour and water were mixed briefly with a plastic paddle to form a slurry, and the cylinder and paddle were then inserted into the viscometer. The slurry was first heated to 50 °C and held at that temperature for 1.5 min, followed by heating at a rate of 12 °C/min to 95 °C, holding for 2.5 min, then cooling to 50 °C at a rate of 12 °C/min, all while continuously stirring and measuring viscosity. A thermogram produced by the viscometer software showed the changes in viscosity over the entire cycle duration, as well as summary statistics of peak viscosity, trough viscosity, breakdown (peak–trough viscosity), final viscosity, setback (final–peak viscosity), peak time, and pasting temperature.

Thermal properties of rice samples such as onset, peak, and gelatinization temperatures (GT) at the conclusion, were measured using a differential scanning calorimeter operating in modulating mode (DSC-Q100, TA Instruments, New Castle, DE, USA). Approximately 4 mg of rice flour were placed into an aluminum DSC pan to which 8 μL of distilled water was added via a micro syringe. After sealing, the pan was equilibrated at room temperature for 1 h prior to being heated from 25 °C to 120 °C at 10 °C/min.

### 2.5. Cooking Qualities

Optimum cooking duration (OCD) for milled rice was measured using a Ranghino test [26]. In a 250 mL beaker, 100 mL of distilled water was boiled (at 98 ± 1 °C), and 5 g of head rice samples were placed into the boiling water, after which measurement of cooking duration was immediately started. After 10 min, 10 grains of rice were removed at one-minute intervals and pressed between two clean glass plates. It was first determined when at least 90% of the grains no longer had opaque cores or uncooked centers, after which the rice was allowed to simmer for an additional 2 min to ensure complete cooking. The OCD included the additional 2 min simmer. The measurement was performed in triplicate and averaged among the three replications.

The length expansion ratio was calculated as a ratio of the length of cooked grain to that of raw grain, and the width expansion ratio was similarly calculated as the ratio of the width of the cooked rice to the initial width of the raw rice [26]. This procedure was performed in triplicate. Both length and width dimensions of a 100-kernel (approximately 1 g) sample were measured, and subsequently, the rice was cooked for an optimum cooking duration and, with both dimensions after cooking, then measured. Measurements were repeated ten times for each rice sample.

### 2.6. Texture Profile Analysis of Cooked Rice

A texture profile analysis (TPA) of cooked rice from each subsample collected during descriptive sensory analysis (see Section 2.7) was performed using a texture analyzer (TA-XT2i, Stable Micro Systems, Ltd., Godalming, Surrey, UK) with a 5 kg load cell and a cylinder probe of 20 mm diameter. The data were acquired using Texture Exponent 32 (Stable Micro Systems, Ltd.). A two-cycle compression was established on three intact rice kernels placed on a clean flat aluminum base, with the compression probe traveling the distance found to compress the kernels to 70% of their original height. Crosshead pretest, test, and post-test speeds were 0.5 mm/s, 3.0 mm/s, and 0.5 mm/s, respectively. The rice samples were analyzed for four TPA parameters: hardness (*N*), adhesiveness (*N* × sec), cohesiveness, and chewiness [27]. Measurements were repeated ten times for each rice sample.

### 2.7. Descriptive Sensory Analysis

Descriptive sensory analysis of cooked rice was conducted at the University of Arkansas Sensory Science Center (Fayetteville, AR, USA). The protocol used in this study was approved by the Institutional Review Board of the University of Arkansas. Prior to participation, written informed consent was obtained from each panelist.

Nine professionally-trained panelists, each with an average experience of more than 1000 h in evaluating a variety of food products, including cooked rice, participated in the descriptive analysis. Prior to the assessment of the samples, 6 h orientation/training sessions conforming to the Spectrum method (Sensory Spectrum Inc., Chatham, NJ, USA) were conducted. As shown in Supplementary Table S1, a total of 19 sensory characteristics: 2 appearances (degree of whiteness and grain size), 7 aromas/aromatics (starchy, grainy, cardboard/papery, sweet aromatic, metallic, burlap, and floral/minty), 4 basic tastes (sweetness, sourness, bitterness, and saltiness), and 6 textural attributes (manual stickiness, initial cohesion, hardness, crunchy cores, tooth pull, and metallic feeling factor), were defined with their reference samples.

Each milled rice subsample (300 g) was cooked in an electric rice cooker (RC3314W rice cooker, Black and Decker, Beachwood, OH, USA) with a 1:1.8 rice-to-water mass ratio. After being cooked, the rice was allowed to sit for five min, after which the cooked rice samples were mixed and fluffed in the rice cooker, using a plastic fork to ensure homogeneity, dipped using a plastic spoon, then presented to the panelists. Each cooked rice subsample (30 g) was placed in a glass bowl (118 mL), identified with a three-digit code, covered with a glass lid, and presented at approximately 71 °C [18]. Individual panelists were asked to evaluate intensities of individual sensory attributes on a scale ranging from 0 to 15 with 0.1 increments. The test samples were randomly presented to the panelists in a sequential monadic fashion. A 10 min break was allowed between sample presentations. During the break, spring water (Clear Mountain Spring Water, Taylor Distribution, AR) was presented. In order to provide two replicate sensory analyses of the cooked rice samples, the entire test was repeated on a different day.

### 2.8. Statistical Analysis

Statistical analyses were performed using JMP Pro software (version 16.0, SAS Institute Inc., Cary, NC, USA). In order to determine the effect of thickness fraction on physicochemical properties, cooking qualities, and textural properties of rice samples, Student’s *t*-tests were conducted for both individual rice cultivar and combined samples, respectively. In order to understand the effect of thickness fraction on the RVA patterns of rice samples better, a functional principal component analysis (FPCA) on the RVA curve data was conducted [28]. Sensory data were analyzed using a four-way ANOVA that treated “thickness fraction” and “rice cultivar” as fixed effects and “panelist” and “repetition” as random effects. If a significant difference in mean values was indicated by the ANOVA, post hoc multiple pairwise comparisons between variables were performed using Tukey’s HSD test. A statistically significant difference was defined whenever *p* < 0.05.

## 3. Results

### 3.1. Thickness Fraction Process-Induced Mass Distributions of Thin and Thick Rice Kernels

**Figure 1:**
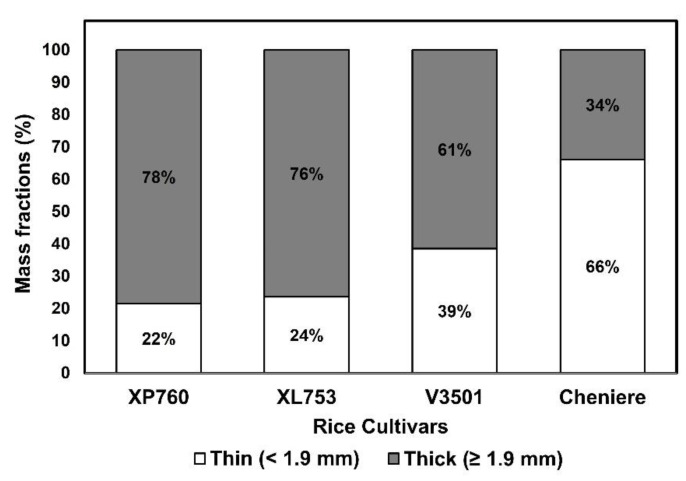
Mass fraction percentages of thin (<1.9 mm) and thick (≥1.9 mm) kernels for each rice cultivar.

The thickness fraction of rough rice resulted in thick fractions ranging from 34% to 78% of bulk rice on a mass basis (Figure 1). A thick kernel range of the bulk rice can vary as a function of rice cultivar and cutoff levels of thickness grading [20,21,29]. For example, Grigg and Siebenmorgen [21] showed a narrower range of thick kernels (>2.0 mm), with values ranging between 67% and 89% of the bulk rice on a mass basis, after thickness fraction was included in the process stream of four long-grain cultivars (CL 151, CL XL729, CL XL745, and Wells). Matsue et al. [29] similarly reported thick kernels (≥1.9 mm) in a narrower range of 85% to 97% in three short-grain rice cultivars (Koshihikari, Nipponbare, and Hinohikari). In the current study, hybrid cultivars XP760 and XL753 exhibited the lowest percentage of thin kernels, with 22% and 24% of thin mass fractions, respectively, while pureline cultivar V3501 exhibited a thin mass fraction of 39%, nearly two times higher than that of the two hybrid cultivars. For Cheniere, the mass distribution of the thin-kernel fraction was almost three times higher than that of the hybrid cultivars (66%). Such a high proportion of thin kernels found in the purelines Cheniere and V3501 would represent a non-favorable scenario in a realistic setting if thickness fraction were to be applied since the thinner kernels would then have to be removed. Different mass distributions of thickness fractions were previously reported across various hybrid and pureline long-grain rice cultivars. Similar to our findings, Jannasch et al. [30] reported that RoyJ, a pureline cultivar, was comprised of a greater proportion of thinner kernels (<1.88 mm), while XL756, a hybrid cultivar, exhibited a greater proportion of thicker kernels (>1.88 mm) after thickness fraction was applied. These results suggest that variations in kernel thickness among cultivars should be taken into consideration when thickness fraction is included in the rice processing stream. Moreover, since environmental conditions such as soil composition and fertilization management may have an even greater impact on kernel thickness distribution than the cultivar effect [22], cultivation conditions within rice cultivars should also be considered even though genetic differences among cultivars can explain some kernel thickness variation distribution.

### 3.2. Effect of Thickness Fraction on Physical Properties of Rice Samples

**Figure 2:**
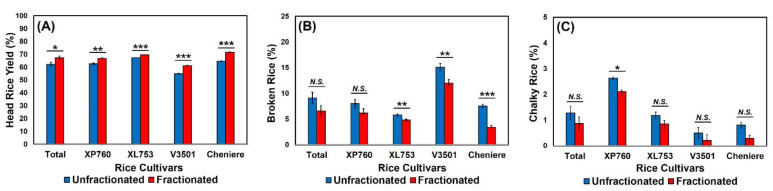
The effects of thickness fraction on (**A**) head rice yield, (**B**) broken rice percentage, and (**C**) chalky rice percentage for each rice cultivar. Error bars represent standard errors of the means. *N.S.* represents no significant difference (*p* > 0.05). *, **, and *** represent a significant difference at *p* < 0.05, *p* < 0.01, and *p* < 0.001, respectively.

Since head rice yield (HRY) is directly related to economic return for rice producers, it is most likely one of the most important indicators of rice quality. Figure 2 shows the effect of thickness fraction on HRY for the four long-grain cultivars: XP760 (*p* = 0.002), XL753 (*p* < 0.001), V3501 (*p* < 0.001), and Cheniere (*p* < 0.001). Across all the evaluated cultivars, fractionated rice samples showed a significantly higher HRY value when compared to an unfractionated rice sample (*t* = −2.65, *p* = 0.02). These results agree with the findings of Grigg and Siebenmorgen [22], who showed that thicker kernels produced dramatically greater HRYs than unfractionated rice, with the reduced number of HRYs in unfractionated rice mainly explained by the susceptibility of thin kernels to breakage during the milling process.

The impact of thickness fraction on the percentage of broken rice kernels was found to vary with rice cultivars. For cultivars XL753 (*p* = 0.007), V3501 (*p* = 0.003), and Cheniere (*p* < 0.001), the percentages of broken kernels were significantly higher in unfractionated rice than in fractionated rice. Hybrid cultivar XP760 exhibited the same numerical trend, although the trend was not statistically significant (*p* = 0.10), probably due to the lower mass percentage of thin kernels compared to those of Cheniere and V3501. Previous research has demonstrated that thin kernels reach a target SLC during milling in a shorter time than thick kernels [3,4]. Therefore, if unfractionated bulk rice is milled for the same duration without any distinction of kernel thickness, the thin kernels would be more likely to be over-milled, resulting in higher susceptibility to breakage and a further reduction in rice yields [3]. Since rice is often consumed as milled, intact kernels, a higher percentage of broken kernels in the unfractionated portion can decrease the economic value of the bulk rice. According to Mukhopadhyay and Siebenmorgen [31], broken kernels are typically worth only 60–80% of the market value of head rice.

As with broken kernels, rice chalkiness was also impacted by the thickness fraction process, depending on the rice cultivar. The thickness fraction process was found to affect the percentage of chalky kernels in cultivar XP760 (*t* = 2.86, *p* = 0.046), but not in other cultivars: XL753 (*p* = 0.21), V3501 (*p* = 0.28), and Cheniere (*p* = 0.06). An association between the number of thin kernels and chalkiness in rice was previously demonstrated [32], with the presence of immature thin kernels in the unfractionated portion increasing the proportion of grains with this undesirable characteristic. Since chalky kernels are more prone to fissures and breakage during milling, the larger number of chalky kernels may also explain the lower HRY values found in the unfractionated portions [2]. Zhao and Fitzgerald [33] established that a ~1% decrease in chalkiness translates to a ~1% increase in HRYs. From a consumer acceptability standpoint, the lower number of chalky kernels in thickness-graded rice could positively impact acceptance of appearance and the cooking and textural qualities of rice [33,34].

As shown in Table 1, the degree of whiteness and discoloration of seeds were not significantly different between unfractionated and fractionated rice (*p* > 0.05 for all). Since Mathews et al. [1] reported that across six different lots of long-grain rice, thinner fractions of rice were perceptively darker than the thicker fractions, and the unfractionated portion included both thick and thin kernels, a higher amount of discoloration would be expected in unfractionated rice. However, in the current study, rice color seemed unaffected by the presence or absence of thinner kernels.

### 3.3. Effects of Thickness Fraction on Chemical Properties of Rice Samples

**Figure 3:**
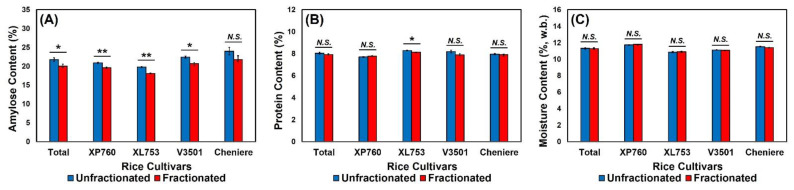
The effects of thickness fraction on (**A**) amylose content, (**B**) protein content, and (**C**) moisture content for each rice cultivar. Error bars represent standard errors of the means. *N.S.* represents no significant difference (*p* > 0.05). * and ** represent a significant difference at *p* < 0.05 and *p* < 0.01, respectively.

Overall, fractionated rice exhibited a significantly lower content of insoluble amylose compared to unfractionated rice (*t* = 2.50, *p* = 0.02). Such difference was also observed in each rice cultivar, except for Cheniere (*p* = 0.12), i.e., XP760 (*p* = 0.006), XL753 (*p* = 0.001), and V3501 (*p* = 0.04) (Figure 3A). Matsue et al. [29] and Siebenmorgen et al. [2] reported differences in amylose content as a function of rice thickness fraction; more specifically, the amylose–amylopectin ratio of rice was found to increase with thickness increments. Since amylose content is positively correlated with intra-panicle rice kernel weight, immaturely-thin kernels would be expected to have lower amylose content than completely mature thick kernels [2]. In the current study, since unfractionated rice contained both thick and thin kernels, the results obtained were probably the consequences of an additive effect of the overall kernel thickness compared to the fractionated portion that was composed only of thick kernels. Lower amylose contents in rice are correlated to a larger proportion of rapidly digestible starch (RDS) [35], and consumption of foods containing RDS, compared to those containing slowly digestible starch (SDS), was shown to increase blood glucose levels quickly after consumption, which might be associated with the development of obesity, insulin resistance, and type 2 diabetes [35,36,37]. This suggests that the higher content of amylose in unfractionated rice may give it a nutritional advantage compared to rice with thicker kernels only. Higher amylose contents in rice have also exhibited better performance in particular applications such as 100% rice bread, where amylose content was positively correlated with final loaf volume [38]. The sensory quality of cooked rice could also be influenced by its amylose content. While rice amylose content is positively correlated with hardness and negatively correlated with the stickiness of cooked rice [39], the relationship between amylose content and consumer preference is not so straightforward. Group differences such as cultural background can also play an important role with respect to rice preference. For example, consumers from countries such as Myanmar, Sri Lanka, and the United States typically favor cooked rice with higher amylose content [39,40], compared to those from other countries such as Korea or Japan that may prefer the eating qualities of thickness-graded rice [41,42].

Similar to its behavior with respect to amylose content, fractionated rice also exhibited lower protein content than unfractionated rice in cultivar XL753 (*t* = 3.97, *p* = 0.02), while such differences were not observed in other cultivars: XP760 (*p* = 0.17), V3501 (*p* = 0.11), and Cheniere (*p* = 0.65) (Figure 3B). Matthews et al. [43] found that, after size fraction, thinner kernels exhibited higher protein content than thicker kernels. Siebenmorgen et al. [2] also observed a positive correlation between protein content and immature thin rice kernels [2,44]. Even though rice is one of the cereals with the lowest protein content, the net protein utilization of rice is the highest among the cereal grains [45], so the higher amount of protein in unfractionated rice would also pose an advantage from a nutritional standpoint. For moisture contents, no significant effects of thickness fraction were observed in the four rice cultivars: XP760 (*p* = 0.56), XL753 (*p* = 0.73), V3501 (*p* = 0.23), and Cheniere (*p* = 0.12), as shown in Figure 3C.

**Figure 4:**
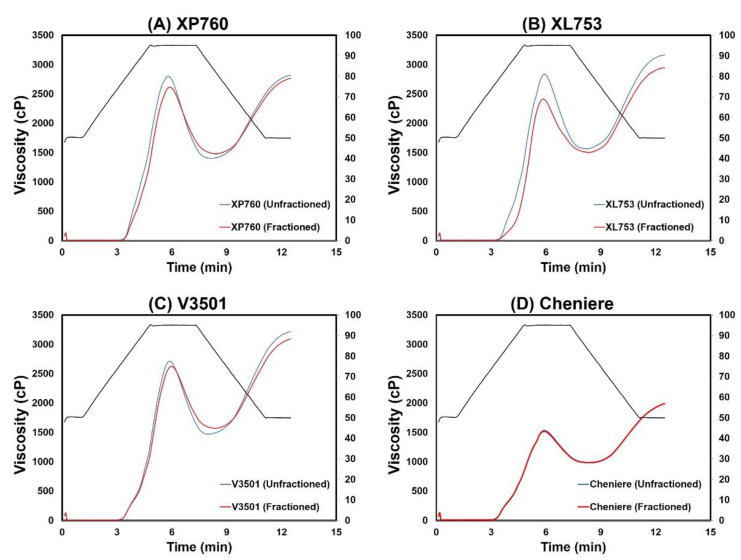
Rapid visco analyzer (RVA) pasting profiles of unfractionated (blue line) and fractionated (red line) rice samples for each rice cultivar.

Figure 4 depicts the RVA pasting profiles of both unfractionated and thickness-fractionated portions of the four long-grain cultivars evaluated. Generally speaking, when visually inspecting pasting profiles, it can be seen that thickness-fractionated rice exhibits a lower viscosity profile than unfractionated rice for all of the long-grain rice cultivars, although the effect for Cheniere was less pronounced. The effects of thickness fraction on various viscosity parameters were found to vary with rice cultivars (Table 2). Hybrids XP760 and XL753 exhibited a significant reduction by approximately 6% and 15%, respectively, in their peak viscosities when thickness fractionating was applied. Wadsworth et al. [32] also reported higher peak viscosities in unfractionated rice compared to thickness-graded rice of a long-grain rice variety (Starbonnet), indicating that the effects of the various fractions on peak viscosity are additive. However, peak viscosities of purelines Cheniere and V3501 seemed to be unaffected by the thickness fraction process (*p* > 0.05), possibly because the pureline cultivars had a significantly larger mass distribution of thinner kernels in the unfractionated portion compared to that in the hybrid cultivars. Interactions between thickness fraction and rice cultivar were also observed with respect to final viscosity and setback (Table 2). For cultivars XL753 and V3501, the final viscosities showed a significant decrease after thickness fractioning, while an opposite trend was seen for the pureline Cheniere, where the final viscosity was significantly higher in the fractionated portion compared to the unfractionated portion of the same cultivar. These results suggest that genotypic variance in final viscosities and setbacks must be considered when a thickness fraction process is included, and selection of a cultivar for further size fraction should be performed on the basis of the functional properties and end applications of the rice.

**Figure 5:**
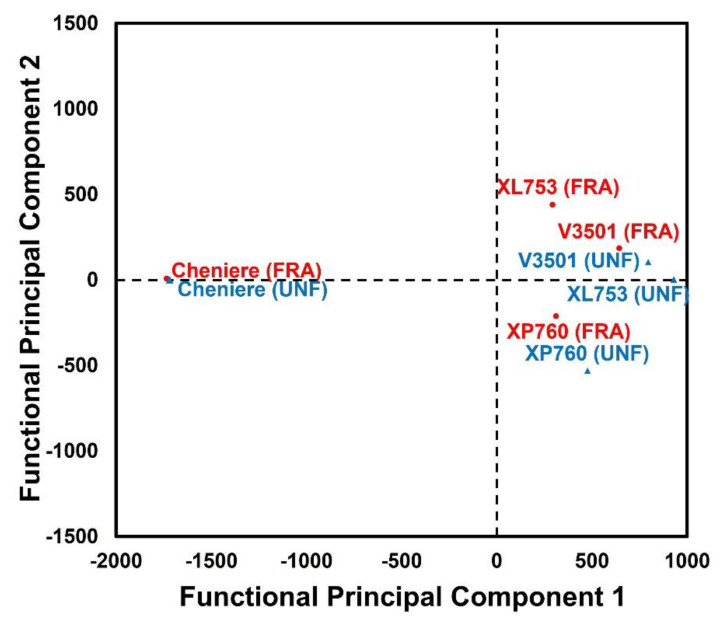
A biplot of the functional principal component analysis (FPCA) based on the rapid visco analyzer (RVA) pasting profiles of unfractionated (UNF) and fractionated (FRA) samples of the four rice cultivars: XP760, XL753, V3501, and Cheniere. The first two functional principal components (FPC) accounted for 99.04% of the total variances, with FPC 1 and FPC 2 explaining 92.70% and 6.34%, respectively.

As previously indicated, RVA profile parameters such as peak viscosity, breakdown, final viscosity, setback, peak time, and pasting temperature are traditionally used to reflect the characteristics of rice flour [46,47]. While such a parameter-based analysis approach can be quite helpful, it reduces the potential value of the RVA full-pattern profile, suggesting that multivariate analysis can be used to obtain a better common pattern from complex data [48] such as the RVA type. More specifically, functional principal component analysis (FPCA), a technique for exploring major sources of variation in a sample of random curves [28], could be useful for this purpose. Figure 5 shows the projection of the RVA profile data onto the first two FPCA components, where the first two functional principal components can be seen to explain as much as 99.0% of the total variation. Genotype was a major contributor to the separation of the rice samples along FPC1, and the Cheniere cultivar (both fractionated and unfractionated) was grouped away from the other cultivars, with high negative loading on FPC1. The FPC2 discriminated the rice samples based on their fractionation condition, and fractionated cultivars XL753, XP760, and V3501 exhibited higher positive loadings on FPC2 compared to the same cultivars when unfractionated. Cheniere, on the other hand, exhibited close-together loadings on FPC2 for both unfractionated and fractionated conditions. These results support the above parameter-based analysis findings in which genotypic differences seemed to play a vital role in determining the impact of size fraction on rice flour viscosity.

As shown in Table 3, onset gelatinization temperature increased up to 1 °C for XP760 and up to 0.9 °C for XL753 when the rice samples were fractionated, although the incremental trends in onset gelatinization temperatures for both Cheniere and V3501 cultivars were not statistically significant (*p* > 0.05). Peak gelatinization temperatures did not differ as a function of thickness fraction, regardless of the cultivar: XP760 (*p* = 0.07), XL753 (*p* = 0.06), V3501 (*p* = 0.76), and Cheniere (*p* = 1.00). Similarly, Siebenmorgen et al. [2] and Wadstworth [32] also found that thickness fraction had little effect on gelatinization temperatures. No significant differences in terms of end gelatinization temperatures and gelatinization enthalpies were found between the unfractionated and fractionated portions for any of the cultivars (*p* > 0.05 for all), although cultivar XL753 exhibited a lower end gelatinization temperature when thickness fraction was applied (*p* = 0.049).

### 3.4. Effects of Thickness Fraction on Cooking Qualities of Rice Samples

**Figure 6:**
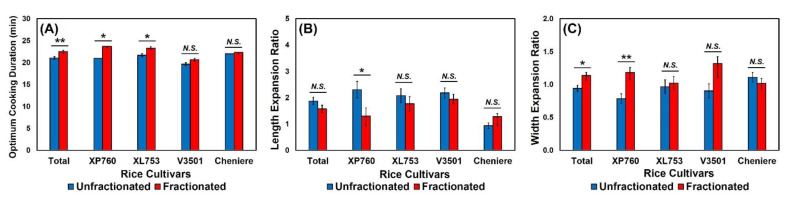
The effects of thickness fraction on (**A**) optimum cooking duration, (**B**) length expansion ratio, and (**C**) width expansion ratio for each rice cultivar. Error bars represent standard errors of the means. *N.S.* represents no significant difference (*p* > 0.05). * and ** represent a significant difference at *p* < 0.05 and *p* < 0.01, respectively.

Rice cooking qualities were evaluated in terms of optimum cooking duration (OCD) and grain elongation during cooking. As shown in Figure 6, the OCD was significantly different between unfractionated and fractionated rice samples in hybrids XP760 (*p* = 0.02) and XL753 (*p* = 0.02). For XP760, the OCD increased from 21 min to 24 min and for XL753 from 22 min to 23 min when rice was fractionated. Even though such trends were also observed in purelines V3501 (*p* = 0.10) and Cheniere (*p* = 0.42), the differences were not statistically significant. Bhattacharya and Sowbhagya [49] and Oko et al. [50] revealed a positive correlation between gelatinization temperature and OCD, and since thickness-fractionated rice exhibited higher onset and peak gelatinization temperature values than the unfractionated rice, this was reflected in longer cooking durations. Grain thickness was also shown to have a significant effect on the cooking duration of rice [51,52] because of a quicker diffusion of moisture in thinner grains that were present exclusively in the unfractionated portion in this study. The cooking time of rice has been a significant determinant of the price at the retail level for consumers in various countries. For example, urban and rural consumers in the Philippines, Indonesia, and Bangladesh seem willing to pay a higher price for rice that disintegrates faster and requires a shorter cooking time [53,54], suggesting an advantage for unfractionated rice in terms of cooking quality. More recently, Calingacion et al. [40] pointed out the possible implications of longer cooking times on the environment, with each minute fewer of individual cooking time globally representing 2500 years of cooking time saved per day. Thus, shorter cooking times time such as those exhibited by unfractionated rice would lead to significant savings of the fuel and measurable reduction in the carbon footprint of rice [40,55].

The effect of thickness fraction on the length elongation, often used as a good quality indicator in rice, was found to vary with rice cultivars (Figure 6B). While the length expansion ratio was significantly greater when the cultivar XP760 was fractionated compared to its unfractionated status (*p* = 0.049), such differences were not observed in other rice cultivars: XL753 (*p* = 0.45), V3501 (*p* = 0.37), and Cheniere (*p* = 0.35). Conversely, fractionated samples of the cultivar XP760 exhibited significantly higher elongation with respect to girth than that of the unfractionated portion (*p* = 0.008). Since this characteristic is associated with rice bursts during cooking, it is undesirable in terms of rice quality and a non-appealing defect for consumers. Bhattacharya [5] reported that rice that expands in length but not so much in girth is associated with good quality. No significant effects of thickness fraction on the width length ratio were obtained in other rice cultivars: XL753 (*p* = 0.72), V3501 (*p* = 0.10), and Cheniere (*p* = 0.09).

### 3.5. Texture Profile Analysis (TPA)

Textural properties of rice were also used as indicators of rice quality. As shown in Table 4, the thickness fraction process did not affect the hardness, cohesiveness, and chewiness parameters of the rice samples (*p* > 0.05 for all), which is similar to the results reported by Siebenmorgen et al. [2], where no significant differences were found with respect to hardness among different rice kernel thickness. On the other hand, thickness fraction decreased the stickiness of rice samples for cultivar Cheniere (*p* = 0.02), but not for other cultivars: XP760 (*p* = 0.91), XL753 (*p* = 0.50), and V3501 (*p* = 0.19). Greater stickiness values were previously correlated with lower amylose contents and lower protein contents [26,56]. In this study, fractionated rice had lower amylose and protein contents (Figure 3), exhibiting lower stickiness values than unfractionated rice. Siebenmorgen et al. [2] reported that the higher the water uptake ratio, the greater the stickiness, possibly supporting these results. Previous studies showed that rice cultivars with high stickiness receive good acceptance by Asian consumers [41,42,57,58], giving a sensory advantage to unfractionated rice. However, an opposite relationship is observed in other areas such as the United States, where consumers prefer harder and less sticky rice [14,16,41,42].

### 3.6. Descriptive Sensory Analysis

For all sensory attributes evaluated, there were no significant interactions between thickness fraction and rice cultivar (*p* > 0.05 for all) (Table 5). There were no significant effects of thickness fraction on sensory attribute intensities of the four rice samples (*p* > 0.05 for all), as shown in Table 5 and Supplementary Table S2. No significant effects of rice cultivar on intensities of sensory attributes, except for metallic feeling factor (*F* = 2.99, *p* = 0.03), were observed. Cultivar V3501 exhibited higher intensities of metallic feeling factor than XP760 (*p* = 0.04).

The results from the descriptive sensory analysis indicate that untrained consumers may be unable to detect differences in appearance, flavor, or textural attributes of milled rice samples when the thickness fraction was applied. Since the physicochemical properties of rice, particularly amylose and protein contents, were affected by thickness fraction, sensory properties were also expected to be impacted, but such effects were not observed in this study. Most studies that have explored relationships between chemical properties and sensory characteristics of rice used samples with significantly large variations in their chemical properties [13,59]. For example, Champagne et al. [13] found that the sensory characteristics of 17 rice cultivars varied significantly depending on the cultivars’ amylose and protein content, and, more specifically, that grain flavor, roughness, and hardness attributes were significantly impacted by the rice’s chemical properties. The amylose contents on Champagne et al.’s rice samples ranged from 1.0% to 26.3%, and the protein contents ranged from 5.7% to 12.0% [13]. In this study, the largest percentage differences between unfractionated and fractionated rice samples were 2.2% for amylose content (Cheniere) and 0.33% for protein content (Cheniere), and these differences were probably not large enough to produce perceptible changes in the sensory characteristics as a function of thickness fraction.

The thickness cutoff chosen for the study could also have played a role in the lack of significance of the sensory attributes. In the previous study conducted by Matsue et al. [29], for grains thinner than 1.9 mm, the thinner the grain, the poorer the appearance, the less stickiness, and the lower the acceptability, while sensory acceptability for grains thicker than 2.0 mm did not vary with grain thickness. Thus, since this study employed a cutoff of 1.9 mm, a value based on what would be likely to occur in a realistic scenario in the rice industry, this could explain the small variation with respect to sensory attributes observed.

A type of reference scale used in the descriptive sensory analysis, i.e., the universal scale, could have resulted in the lack of significant difference between unfractionated and fractionated rice samples with respect to aromas/aromatics. The universal scale has often been used as a frame of quantitative reference for a variety of food samples, including cooked rice, because it provides absolute intensity values of sensory attributes, thereby comparing the specific attribute intensities across any category of product samples. However, the primary disadvantage of the universal scale is that panelists may use a narrow range of the entire scale when test samples have either lower or higher attribute intensities on the scale, resulting in a lack of significant difference between the test samples [60,61]. In fact, Jarma Arroyo and Seo [61] showed that the universal aromatic scale using three reference samples across product categories (i.e., crackers, applesauce, and orange juice concentrate) was less sensitive to differentiating cooked rice samples than the rice aromatic scale using three reference sample of cooked rice in the descriptive sensory analysis of cooked rice samples that typically exhibit weak flavors. Therefore, it would be interesting to conduct a further study aimed at determining whether aroma/aromatic differences between unfractionated and fractionated rice samples could be observed when performing descriptive sensory analysis using cooked rice-based reference scales (e.g., rice aromatic scale).

The inclusion of thickness fractions in the process stream would imply the need for logistical changes and an associated increase in production costs for rice-producing companies. By providing a better understanding of how thickness fraction impacts physicochemical properties, cooking qualities, and sensory aspects of rice, this study should help rice producers and processors make informed decisions related to the advantages and disadvantages of including this step in the process flow.

## 4. Conclusions

This study demonstrated the effects of thickness fraction on the physicochemical properties, cooking qualities, and sensory aspects related to four long-grain rice cultivars. From a milling and physical quality perspective, since thickness-graded rice exhibited significantly higher yields as well as fewer broken and chalky kernels, the economic return from using thickness fraction would be enhanced. In addition, while the resulting thinner kernels could offer additional profits by use in other applications such as flour, the reduction in amylose or protein content in fractionated rice may impose a dilemma in terms of how the rice’s nutritional value might seem to be deteriorated by this process. The cooking qualities of cooked rice were also negatively impacted by thickness fraction (i.e., requirement for longer cooking duration and greater width expansion), and since these characteristics are usually not very appealing to some consumers, the economic value of rice treated in this way may be decreased. Rice processors would therefore need to justify the inclusion of thickness fractions in their operations based on the advantages and disadvantages discussed in this study.

## Figures and Tables

**Table 1:** Mean values (± standard deviation) of the rice subsamples with respect to color characteristics as a function of rice cultivar and thickness fraction.

Parameter	Total		XP760		XL753		V3501		Cheniere	
	UNF	FRA	UNF	FRA	UNF	FRA	UNF	FRA	UNF	FRA
Whiteness (L*)	70.8 (±0.8)	70.6 (±0.7)	71.9 (±0.5)	71.6 (±0.3)	70.6 (±0.5)	70.4 (±0.4)	69.9 (±0.1)	70.0 (±0.4)	70.9 (±0.2)	70.5 (±0.4)
Discolored area (%)	10.5 (±7.8)	11.6 (±6.1)	3.3 (±5.8)	3.3 (±5.8)	6.3 (±6.5)	13.3 (±1.5)	20.0 (±3.0)	14.3 (±3.5)	12.3 (±2.9)	15.3 (±4.5)

UNF and FRA represent unfractionated and fractionated rice samples, respectively. No significant difference between unfractionated and fractionated rice samples was observed in each rice cultivar with respect to whiteness or discolored area (*p* > 0.05).

**Table 2:** Mean values (± standard deviation) of the rice subsamples with respect to pasting properties as a function of rice cultivar and thickness fraction.

Parameter	Total		XP760		XL753		V3501		Cheniere	
	UNF	FRA	UNF	FRA	UNF	FRA	UNF	FRA	UNF	FRA
Peak Viscosity (cP)	2464.0 a (±585.7)	2294.0 a (±484.1)	2807.7 a (±97.1)	2622.0 b (±20.0)	2886.7 a (±45.1)	2440.3 b (±23.7)	2650.0 a (±133.0)	2611.7 a (±16.3)	1511.7 a (±15.6)	1502.0 a (±46.5)
Trough Viscosity (cP)	1309.8 a (±314.4)	1366.9 a (±241.7)	1402.0 a (±39.6)	1384.0 a (±92.0)	1558.7 a (±9.9)	1537.0 a (±39.6)	1480.3 b (±10.3)	1556.7 a (±15.3)	798.3 b (±6.8)	990.0 a (±2.6)
Breakdown Viscosity (cP)	1154.2 a (±288.2)	927.1 a (±282.9)	1405.7 a (±57.9)	1238.0 a (±91.7)	1328.0 a (±53.7)	903.3 b (±27.3)	1169.7 a (±134.8)	1055.0 a (±1.0)	713.3 b (±14.7)	512.0 a (±47.0)
Final Viscosity (cP)	2761.1 a (±562.1)	2688.8 a (±453.9)	2810.7 a (±54.2)	2724.0 a (±39.6)	3179.0 a (±31.6)	2969.3 b (±23.2)	3186.3 a (±39.1)	3089.3 b (±22.1)	1868.3 b (±9.)	1972.7 a (±30.9)
Setback Viscosity (cP)	297.1 a (±206.1)	394.8 a (±179.3)	3.0 b (±45.4)	102.0 a (±39.9)	292.3 b (±30.7)	529.0 a (±15.9)	536.3 a (±97.6)	477.7 a (±15.3)	356.7 b (±7.8)	470.7 a (±26.2)
Peak Time (min)	5.8 b (±0.1)	5.9 a (±0.1)	5.8 a (±0.0)	5.8 a (±0.0)	5.9 a (±0.0)	5.9 a (±0.0)	5.9 a (±0.1)	6.0 a (±0.0)	5.7 a (±0.0)	5.9 a (±0.0)
Pasting Temp (°C)	78.8 a (±0.5)	80.5 a (±3.5)	78.5 a (±0.1)	78.7 a (±0.4)	79.5 b (±0.5)	86.3 a (±0.0)	78.7 a (±0.4)	78.5 a (±0.0)	78.5 a (±0.1)	78.7 a (±0.4)

UNF and FRA represent unfractionated and fractionated rice samples, respectively. Mean values with different letters within a row for each rice cultivar represent a significant difference at *p* < 0.05.

**Table 3:** Mean values (± standard deviation) of the rice subsamples with respect to pasting properties as a function of rice cultivar and thickness fraction.

Parameter	Total		XP760		XL753		V3501		Cheniere	
	UNF	FRA	UNF	FRA	UNF	FRA	UNF	FRA	UNF	FRA
Onset GT (°C)	71.1 a (±0.7)	71.6 a (±1.0)	71.2 b (±0.1)	72.2 a (±0.1)	72.0 b (±0.4)	72.9 a (±0.2)	70.3 a (±0.2)	70.4 a (±0.2)	70.7 a (±0.2)	70.9 a (±0.2)
Peak GT (°C)	76.2 a (±0.9)	76.4 a (±1.1)	76.7 a (±0.1)	76.9 a (±0.1)	77.4 a (±0.2)	77.9 a (±0.2)	75.5 a (±0.3)	75.4 a (±0.0)	75.4 a (±0.2)	75.5 a (±0.1)
End GT (°C)	82.9 a (±1.8)	82.1 a (±2.0)	84.1 a (±0.8)	81.6 a (±3.5)	84.9 a (±0.3)	84.2 b (±0.2)	81.5 a (±0.6)	81.4 a (±0.0)	81.0 a (±0.4)	81.0 a (±0.2)
Enthalpy (J/g)	9.5 a (±2.0)	9.0 a (±1.4)	10.4 a (±1.5)	9.4 a (±0.6)	11.5 a (±0.9)	10.1 a (±1.2)	8.7 a (±1.4)	9.3 a (±0.3)	7.4 a (±1.8)	7.1 a (±1.2)

GT represents gelatinization temperature. UNF and FRA represent unfractionated and fractionated rice samples, respectively. Mean values with different letters within a row for each rice cultivar represent a significant difference at *p* < 0.05.

**Table 4:** Mean values (± standard deviation) of the rice subsamples with respect to textural profile analysis parameters of cooked rice as a function of rice cultivar and thickness fraction.

Parameter	Total		XP760		XL753		V3501		Cheniere	
	UNF	FRA	UNF	FRA	UNF	FRA	UNF	FRA	UNF	FRA
Hardness (*N*)	14.7 (±1.9)	14.4 (±2.1)	14.6 (±2.1)	13.2 (±1.7)	15.2 (±1.7)	15.7 (±2.5)	13.6 (±1.6)	13.4 (±1.4)	15.6 (±1.8)	15.5 (±1.2)
Adhesiveness (*N* × sec)	0.6 (±0.2)	0.5 (±0.2)	0.6 (±0.2)	0.6 (±0.2)	0.7 (±0.3)	0.6 (±0.2)	0.5 (±0.2)	0.4 (±0.2)	0.6 (±0.2)	0.3 (±0.2)
Cohesiveness	0.4 (±0.0)	0.4 (±0.0)	0.4 (±0.0)	0.4 (±0.0)	0.4 (±0.0)	0.4 (±0.0)	0.4 (±0.0)	0.4 (±0.0)	0.4 (±0.0)	0.4 (±0.0)
Chewiness (*N*)	4.4 (±0.9)	4.2 (±1.0)	4.6 (±0.7)	4.0 (±0.9)	4.2 (±0.7)	4.8 (±1.5)	4.3 (±1.1)	3.8 (±0.7)	4.3 (±1.0)	4.2 (±0.6)

UNF and FRA represent unfractionated and fractionated rice samples, respectively.

**Table 5:** *F*-ratios (*p*-value) determined by the four-way analysis of variance, treating thickness fraction and rice cultivar as fixed effects and panelist and session as random effects, with respect to sensory attributes of cooked rice samples.

Attributes	Thickness Fraction (TF)	Rice Cultivar (RC)	TF × RC
Appearance			
Degree of whiteness	0.13 (0.72)	2.06 (0.11)	1.09 (0.36)
Grain size	0.39 (0.54)	1.70 (0.17)	0.56 (0.65)
Aromas/Aromatics			
Starchy	1.05 (0.31)	2.07 (0.11)	0.17 (0.92)
Grainy	1.38 (0.24)	0.60 (0.62)	1.03 (0.38)
Cardboard/Papery	1.24 (0.27)	0.19 (0.90)	1.23 (0.30)
Sweet	3.49 (0.06)	0.39 (0.76)	0.39 (0.76)
Metallic	0.18 (0.67)	0.18 (0.91)	0.18 (0.91)
Burlap	0.53 (0.47)	0.08 (0.97)	1.26 (0.29)
Floral/Minty	2.30 (0.13)	0.71 (0.55)	0.38 (0.77)
Basic Tastes			
Sweetness	1.89 (0.17)	1.07 (0.37)	1.05 (0.37)
Sourness	0.57 (0.45)	0.45 (0.72)	0.45 (0.72)
Bitterness	0.06 (0.81)	0.33 (0.81)	1.45 (0.23)
Saltines	0.84 (0.36)	0.95 (0.42)	1.06 (0.37)
Textural Attributes			
Manual stickiness	0.34 (0.56)	0.67 (0.58)	0.62 (0.61)
Initial cohesion	0.05 (0.83)	2.43 (0.07)	1.69 (0.17)
Hardness	1.32 (0.25)	2.02 (0.11)	0.58 (0.63)
Crunchy cores	1.91 (0.17)	1.90 (0.13)	0.20 (0.90)
Tooth pull	1.30 (0.26)	0.65 (0.58)	0.45 (0.72)
Metallic feeling factor	1.31 (0.26)	2.99 (0.03)	1.35 (0.26)

## Data Availability

The data are not publicly available due to the Institutional Review Board protocol guideline.

## Supplementary Materials

The following are available online at https://www.mdpi.com/article/10.3390/foods11020222/s1, Table S1: Descriptors, definitions, and references for appearance, aroma/aromatics, and textural attributes of cooked rice used in the descriptive sensory analysis. Table S2: Mean values (± standard deviation) of the rice subsamples with respect to sensory attributes as a function of rice cultivar and thickness fraction.
