# Supplementary material for: Effects of Thickness Fraction Process on Physicochemical Properties, Cooking Qualities, and Sensory Characteristics of Long-Grain Rice Samples
Source: Foods. 2022 Jan 14;11(2):222. doi: 10.3390/foods11020222 (PMC8775037; doi:10.3390/foods11020222)
Supplement: Supplementary file 1 [file foods-11-00222-s001.zip › foods-1520794-supplementary.pdf]

Supplementary Table S1. Descriptors, definitions, and references for appearance, aroma/aromatics, and textural attributes of cooked rice used in the descriptive sensory analysis

Descriptors	Definition	References[1] (Intensity)
**Appearances**		
Degree of whiteness	The degree to which the sample is pure white	Ref. D (13.0)<br>Ref. C (6.0)
Grain size	The overall dimensions of the rice kernels in terms of width and length	Ref. D (7.5)
**Aromas/Aromatics**		
Starchy	The aromatic associated with the starch of a particular grain source	UAS[2]
Grainy	A general term used to describe the aromatics of raw or cooked grains, which cannot be tied to a specific grain type	UAS
Cardboard/Papery	The aromatic associated with early stages of oxidation	UAS
Sweet aromatic	The aromatic associated with materials that also have a sweet taste, such as molasses, caramelized sugars, cotton candy, maple syrup, or maltol	UAS
Metallic	The aromatic associated with metals, tinny, or irony	UAS
Burlap	The aromatic associated with burlap	UAS
Floral/Minty	The aromatic associated with a non-specific floral note and sometimes described as minty	UAS
**Basic tastes**		
Sweetness	The basic taste, perceived on the tongue, stimulated by sugars and high potency sweeteners (reference: sucrose solution)	2% (2.0)<br>5% (5.0)<br>10% (10.0)<br>16% (15.0)
Sourness	The basic taste, perceived on the tongue, stimulated by acids such as citric acid (reference: citric acid solution)	0.05% (2.0)<br>0.08% (5.0)<br>0.15% (10.0)<br>0.20% (15.0)
Bitterness	The basic taste, perceived on the tongue, stimulated by substances such as quinine, caffeine, and certain other alkaloids (reference: caffeine solution)	0.05% (2.0)<br>0.08% (5.0)<br>0.15% (10.0)<br>0.20% (15.0)
Saltiness	The basic taste, perceived on the tongue, stimulated by sodium salt, especially sodium chloride (reference: sodium chloride solution)	0.2% (2.0)<br>0.5% (8.5)<br>0.7% (15.0)
**Textural attributes**		
Manual stickiness	The force required to separate the fingers after compressing the sample between the thumb and forefinger ("none" to "much sticky")	Ref. A (5.0)
Initial cohesion	The degree to which the un-chewed sample sticks together ("loose" to "tight")	Ref. B (1.0)<br>Ref. A (3.0)<br>Ref. D (11.0)

Hardness	The force required to compress the sample ("soft" to "hard")	Ref. D (1.0)<br>Ref. A (4.0)<br>Ref. B (5.0)<br>Ref. C (10.0)
Crunchy cores	The amount of crunchy centers perceived in the sample while chewing the sample 4-5 times	Ref. A (5.0)<br>Ref. C (12.0)
Tooth pull	The force required to separate the teeth during mastication by evaluating on the 3rd pull ("none" to "much sticky")	Ref. B (2.0)<br>Ref. A (4.0)
Metallic feeling factor	The aromatics associated with metals, tinny or iron or a flat chemical feeing factor stimulated on the tongue by metal coins	UAS

[1]References A to D = 300 g of raw rice was cooked in an electronic rice cooker (RC3314W, Black & Decker, Beachwood, OH, USA) with a 1:3.5 rice-to-water mass ratio. Specifically, Reference A: Riceland Extra Long Grain Brown Rice (Riceland Foods, Stuttgart, AR, USA) cooked for 35 min; Reference B: Uncle Ben's Converted Brown Rice (Mars Food, McLean, VA, USA) cooked for 25 min; Reference C: Riceland Extra Long Grain Brown Rice (Riceland Foods) cooked for 25 min; and Reference D: Riceland Extra Long Grain Milled Rice (Riceland Foods) cooked for 25 min. Each cooked-rice reference was held in the rice cooker for 5 min and then gently fluffed. Next, 30 g of the cooked rice was placed in a 118-mL glass bowl, covered with a glass lid, and served at approximately 71 °C.

[2]UAS: Universal Aromatic Scale with a modification; soda note in Nabisco Premium Original Saltine Crackers (Mondelez Global LLC, East Hanover, NJ, USA) = 3.0; cooked-apple note in Mott's Natural Applesauce (Mott's LLP, Plano, TX, USA) = 7.0.

Supplementary Table S2. Mean values (± standard deviation) of the rice subsamples with respect to sensory attributes as a function of rice cultivar and thickness fraction

Attributes	Total		XP760		XL753		V3501		Cheniere	
	UNF	FRA	UNF	FRA	UNF	FRA	UNF	FRA	UNF	FRA
**Appearance**										
Degree of whiteness	12.5 (± 0.6)	12.5 (± 0.6)	12.3 (± 0.6)	12.4 (± 0.6)	12.4 (± 0.7)	12.6 (± 0.6)	12.5 (± 0.5)	12.4 (± 0.7)	12.6 (± 0.5)	12.6 (± 0.7)
Grain size	6.7 (± 0.7)	6.8 (± 0.7)	6.7 (± 0.6)	6.9 (± 0.6)	6.7 (± 0.7)	6.6 (± 0.8)	6.5 (± 0.6)	6.7 (± 0.7)	6.9 (± 0.6)	6.8 (± 0.8)
**Aromas/Aromatics**										
Starchy	5.4 (± 0.7)	5.3 (± 0.8)	5.5 (± 0.6)	5.4 (± 0.8)	5.4 (± 0.7)	5.3 (± 0.8)	5.3 (± 0.8)	5.3 (± 0.8)	5.2 (± 0.9)	5.2 (± 0.9)
Grainy	3.8 (± 0.8)	3.7 (± 1.0)	4.0 (± 0.6)	3.7 (± 1.1)	3.9 (± 0.6)	3.7 (± 1.1)	3.7 (± 1.1)	3.7 (± 1.1)	3.7 (± 1.1)	3.8 (± 0.7)
Cardboard/ Papery	2.4 (± 1.4)	2.3 (± 1.5)	2.3 (± 1.6)	2.4 (± 1.5)	2.4 (±1.4)	2.4 (± 1.5)	2.6 (± 1.3)	2.2 (± 1.6)	2.4 (± 1.5)	2.2 (± 1.6)
Sweet	0.1 (± 0.7)	0.0 (± 0.0)	0.2 (± 0.8)	0.0 (± 0.0)	0.2 (± 0.8)	0.0 (± 0.0)	0.0 (± 0.0)	0.0 (± 0.0)	0.2 (± 0.8)	0.0 (± 0.0)
Metallic	1.0 (± 1.5)	1.0 (± 1.6)	0.9 (± 1.6)	1.0 (± 1.6)	0.9 (± 1.6)	1.1 (± 1.7)	1.0 (± 1.6)	0.9 (± 1.6)	1.1 (± 1.6)	1.0 (± 1.6)
Burlap	1.3 (± 1.6)	1.4 (± 1.6)	1.2 (± 1.6)	1.4 (± 1.7)	1.5 (± 1.6)	1.3 (± 1.7)	1.3 (± 1.6)	1.3 (± 1.7)	1.1 (± 1.7)	1.5 (± 1.7)
Floral/Minty	0.8 (±1.4)	0.6 (± 1.2)	0.7 (± 1.4)	0.4 (± 1.0)	0.7 (± 1.4)	0.7 (± 1.4)	0.7 (± 1.4)	0.5 (± 1.3)	0.9 (± 1.5)	0.7 (± 1.4)
**Basic tastes**										
Sweetness	0.2 (± 0.3)	0.2 (± 0.2)	0.2 ± (0.3)	0.3 ± (0.3)	0.2 ± (0.3)	0.2 ± (0.3)	0.3 ± (0.3)	0.2 ± (0.3)	0.3 ± (0.4)	0.2 ± (0.3)
Sourness	0.4 (± 0.4)	0.4 (± 0.4)	0.4 ± (0.4)	0.4 ± (0.5)	0.4 ± (0.4)	0.4 ± (0.5)	0.5 ± (0.5)	0.4 ± (0.5)	0.4 ± (0.6)	0.4 ± (0.5)
Bitterness	0.2 (± 0.3)	0.2 (± 0.3)	0.3 ± (0.5)	0.2 ± (0.4)	0.2 ± (0.3)	0.3 ± (0.5)	0.2 ± (0.3)	0.3 ± (0.4)	0.3 ± (0.4)	0.3 ± (0.3)
Saltines	0.1 (± 0.1)	0.1 (± 0.7)	0.1 ± (0.2)	0.1 ± (0.2)	0.1 ± (0.2)	0.1 ± (0.2)	0.1 ± (0.2)	0.1 ± (0.2)	0.1 ± (0.2)	0.4 ± (1.5)
**Textural attributes**										
Manual stickiness	6.3 (± 1.0)	6.2 (± 1.1)	6.3 (± 0.7)	6.2 (± 1.1)	6.4 (± 1.1)	6.0 (± 0.8)	6.1 (± 1.4)	6.0 (± 1.2)	6.3 (± 0.9)	6.4 (± 1.2)
Initial cohesion	9.3 (± 2.4)	9.3 (± 2.4)	10.1 (± 1.8)	9.4 (± 2.3)	9.1 (± 2.3)	9.4 (± 2.2)	8.9 (± 2.9)	9.3 (± 2.2)	9.1 (± 2.4)	9.0 (± 2.1)
Hardness	2.1 (± 0.8)	1.9 (± 0.7)	1.8 (± 0.5)	1.9 (± 0.7)	2.1 (± 0.8)	1.9 (± 0.6)	2.1 (± 0.8)	1.8 (± 0.6)	2.2 (± 1.0)	2.2 (± 0.9)
Crunchy cores	1.4 (± 1.2)	1.3 (± 1.2)	1.3 (± 1.0)	1.3 (± 1.3)	1.5 (± 1.3)	1.4 (± 1.3)	1.3 (± 1.2)	1.1 (± 1.2)	1.6 (± 1.2)	1.4 (± 1.1)
Tooth pull	3.5 (± 0.9)	3.6 (± 0.9)	3.4 (± 0.9)	3.7 (± 0.8)	3.5 (± 0.9)	3.5 (± 1.0)	3.4 (± 0.9)	3.6 (± 1.0)	3.7 (± 0.8)	3.7 (± 0.9)

Metallic feeling factor	1.9 (± 2.3)	2.0 (± 2.4)	1.9 ± (2.4)	2.0 ± (2.5)	1.9 ± (2.2)	2.1 ± (2.5)	2.3 ± (2.7)	2.1 ± (2.6)	1.9 ± (2.3)	2.1 ± (2.6)

UNF and FRA represent unfractionated and fractionated rice samples, respectively.
